# Heading for the Hills: Risk Avoidance Drives Den Site Selection in African Wild Dogs

**DOI:** 10.1371/journal.pone.0099686

**Published:** 2014-06-11

**Authors:** Craig R. Jackson, R. John Power, Rosemary J. Groom, Emmanuel H. Masenga, Ernest E. Mjingo, Robert D. Fyumagwa, Eivin Røskaft, Harriet Davies-Mostert

**Affiliations:** 1 Department of Biology, Norwegian University of Science and Technology, Trondheim, Norway; 2 Department of Economic Development, Environment, Conservation and Tourism, North West Provincial Government, Mmabatho, South Africa; 3 Department of Zoology, University of Johannesburg, Johannesburg, South Africa; 4 African Wildlife Conservation Fund, Chishakwe Ranch, Zimbabwe; 5 Tanzania Wildlife Research Institute, Arusha, Tanzania; 6 Endangered Wildlife Trust, Johannesburg, South Africa; 7 Department of Zoology, Wildlife Conservation Research Unit, Recanati-Kaplan Centre, Oxford University, Oxford, United Kingdom; University of Tasmania, Australia

## Abstract

Compared to their main competitors, African wild dogs (*Lycaon pictus*) have inferior competitive abilities and interspecific competition is a serious fitness-limiting factor. Lions (*Panthera leo*) are the dominant large carnivore in African savannah ecosystems and wild dogs avoid them both spatially and temporally. Wild dog young are particularly vulnerable and suffer high rates of mortality from lions. Since lions do not utilize all parts of the landscape with an equal intensity, spatial variation in lion densities can be exploited by wild dogs both during their general ranging behaviour, but more specifically when they are confined to a den with vulnerable young. Since patches of rugged terrain are associated with lower lion densities, we hypothesized that these comparatively safe habitats should be selected by wild dogs for denning. We investigated the relationship between the distribution of 100 wild dog den sites and the occurrence of rugged terrain in four wild dog populations located in Tanzania, Zimbabwe and South Africa. A terrain ruggedness index was derived from a 90 m digital elevation model and used to map terrain ruggedness at each site. We compared characteristics of actual and potential (random) den sites to determine how wild dogs select den sites. The distributions of wild dog dens were strongly associated with rugged terrain and wild dogs actively selected terrain that was more rugged than that available on average. The likelihood of encountering lions is reduced in these habitats, minimizing the risk to both adults and pups. Our findings have important implications for the conservation management of the species, especially when assessing habitat suitability for potential reintroductions. The simple technique used to assess terrain ruggedness may be useful to investigate habitat suitability, and even predict highly suitable denning areas, across large landscapes.

## Introduction

Habitat selection allows animals to select specific habitat attributes which allow them to increase their fitness [1]. This behavioural process may be particularly evident during the breeding season. Since reproductive success is vital for population persistence, it places a strong selective force on mechanisms and strategies to optimize the survival of young. Many mammalian carnivores require a den to successfully rear offspring [2]–[3]. The locations of den sites are therefore not randomly dispersed across a landscape but are instead often selected based on factors such as food availability [4]–[5], shelter from extreme weather [6] or predator evasion [7]–[8].

Endangered African wild dogs (*Lycaon pictus*) live in highly social packs which cooperate to feed and protect the dominant pair’s offspring [9]. Wild dog packs reproduce once a year and spend approximately three months at a den before pups become mobile [9]. Larger packs are generally more reproductively successful than smaller ones and this is attributed to a greater hunting efficiency and defence of young and kills [10]–[12]. The annual reproductive event is thus a vital component of wild dog ecology that strongly influences pack survival and population persistence.

Among the large African carnivores, wild dogs are far smaller than their main competitors, lions (*Panthera leo*) and spotted hyaenas (*Crocuta crocuta*) [13]. Lions account for as much as 12% of adult and 31% of wild dog pup mortality [14]. Although spotted hyaenas may steal wild dog kills [15], they account for only 4% of adult and 6% of pup mortality [14]. Lions impose a far greater risk to wild dogs and are avoided whenever they are encountered or detected [16]. Although wild dogs can rapidly cover large distances (spatial avoidance; [17]), this mechanism facilitating coexistence is negated during the denning period when packs are confined to den sites. Wild dog mortality rates are highest during the first few months of life [9] and den site selection is thus an important driver of reproductive success.

Lion densities differ in space and as the dominant large carnivore their densities are positively correlated to prey density [18]. Certain landscape features may therefore not be favoured by lions due to a lower abundance of preferred prey species (e.g. [19]) or where topographical and vegetation characteristics make hunting more challenging for the ambush predators [20]–[21]. This causes a heterogeneous risk landscape allowing wild dogs to behaviourally mediate the risk of interspecific encounters by the selection of lower risk habitats. Consequently, wild dog densities tend to be inversely correlated to those of lions [18].

Variations in the two species’ densities are strongly associated with specific habitat types. For example, in the Kruger National Park, South Africa, important prey species for lions, such as buffalo (*Syncerus caffer*), are associated with open savannah landscapes [18]. These prey-rich landscape types were the most preferred habitat types for lions [18]. In contrast, lions avoided hilly and mountainous terrain which were significantly more preferred by wild dogs and ranked as wild dogs’ most preferred habitat types [18]. Not only were these rugged habitat types ranked as the least preferred by lions, but also by spotted hyaenas [18]. Patches of rugged terrain in the form of hills, ridges or inselbergs thus have the potential to provide wild dog populations with respite from interspecific competition [20].

Given the variation in risk based on habitat types, we hypothesized that wild dogs actively select rugged habitat for denning as part of their risk avoidance strategy. We tested our hypothesis with den site location data from four wild dog populations located in Tanzania, Zimbabwe and South Africa. Selected habitat features are those utilised disproportionately more often than their general availability [22]. To assess whether wild dogs actively select rugged terrain in which to den, we created three random contrast locations per den site, located within the same study area. These locations provide an indication of available but unutilised habitat. Factors influencing den site selection are well studied in other large canids such as wolves (e.g. [23]–[25]) but, excepting for a single recent study on wild dogs [26], are entirely lacking for the species. More specifically, the potential role of rugged terrain for successful reproduction in wild dog populations has never been considered before and is of potential importance for the conservation management of this endangered carnivore.

## Methods

### Study Areas

We used wild dog den site locations from four different ecosystems where lion populations were present in all study locations. The Loliondo Game Controlled Area (LGCA) is situated within the eastern part of the Serengeti Ecosystem in northern Tanzania and is bordered to the west by Serengeti National Park. The area is 4000 km^2^ in size with a population of approximately 130 wild dogs in 8–10 packs [27]. A large part of the LGCA landscape is undulating and hilly. Vegetation types vary greatly within LGCA and range from open woodland to short grass plains. Dominant species include *Acacia drepanolobium* on black cotton soils, high altitude forests of *Juniperus procera*, while the long grass plains are characterised by *Acacia gerardii*, *Rhus natalensis*, *Euclea divinorum*, and *Acacia hockii* tree species [28].

The 3450 km^2^ Savé Valley Conservancy (SVC) is situated in the semi-arid south-east of Zimbabwe and is largely dominated by mopane (*Colophospermum mopane*) shrub or woodland. There are at least 11 known wild dog packs in the SVC, totalling approximately 110 adult and yearling wild dogs. The wild dog population has been studied in the SVC since 1996.

The De Beers Venetia Limpopo Nature Reserve (VLNR) is a 316 km^2^ privately owned reserve located in Limpopo Province in the north of South Africa. The reserve is low-lying with a semi-arid climate and falls within the Limpopo Rugged Bushveld and Musina Mopane Bushveld vegetation types of the savannah biome [29]. The naturally occurring lion population remained relatively stable (∼18 individuals) during the period of this study. A reintroduced population of wild dogs fluctuated between 11–27 individuals. The reserve is enclosed by ‘predator-proof’ electrified perimeter fencing.

The fourth study population, which fluctuated between 5 and 56 individuals in 1–6 wild dog packs, was located in Madikwe Game Reserve (MGR). The 620 km^2^ protected area is situated in the semi-arid north-west region of South Africa. It is located within the savannah biome and vegetation types are classified as Dwarsberg-Swartruggens Mountain Bushveld, Madikwe Dolomite Bushveld and Dwaalboom Thornveld [29].

### Den Site Location Records

Wild dogs often shift the location of their den during the course of the denning period. We only used the first den site locations each season, as this is selected prior to the birth of offspring. Selection of secondary den sites would be limited to some degree by the young pups which would often need to be carried by adults, thus potentially limiting the distance from the initial location. Dens were located by researchers and locations recorded using hand held global positioning systems (GPS). A total of 19 den sites (2005–2009) were used for LGCA, 57 den sites for SVC (2005–2013), 6 den sites for VLNR (2002–2007) and 18 den sites for MGR (1998–2013).

### Random Contrast Location

To determine the extent of variation in terrain ruggedness and the types of habitat available to wild dog packs within each study area, three random locations were generated per known den site in ArcMap 10.2 (ESRI, Redlands, CA, USA). Movement data (not shown) indicated that the majority of study areas were traversed by wild dog packs outside of the denning period. Thus the majority of study areas were theoretically available for denning, although at a finer scale suitable den sites would be required. Packs typically use burrows excavated by aardvarks *Orycteropus afer* or other partly fossorial mammals [13], or caves and crevices in rocky areas [20].

### Terrain Ruggedness Index

To assess the distribution of rugged terrain we used a 90 m digital elevation model (DEM). A spatial filter was applied to determine the standard deviation around each focal grid cell. Since each grid cell value is altitude, neighbouring grid cells in flat areas have little or no difference in altitude. In rugged areas, neighbouring grid cells differ. This difference can be quantified by calculating the standard deviation around each grid cell to provide a terrain ruggedness index (TRI). For this analysis, we selected a grid cell neighbourhood of 3×3 grid cells (270 m×270 m) around each focal grid cell from which the TRI value was calculated. The resulting TRI values reflect fine scale variation in altitudes, not absolute altitude values, and are therefore comparable across ecosystems despite variations between their regional mean altitudes. TRI values for all grid cells within a 100 m, 250 m, and 500 m radius of den sites were extracted and contrasted with TRI values within the same radii from random contrast sites. Using SigmaPlot version 12 (Systat Software Inc.), a Kruskal-Wallis one way analysis of variance (ANOVA) on ranks was used to test for differences between the six groups within each study area. Dunn’s method, a multiple comparison procedure, was used to identify specific groups that differed from one another (significance when P = <0.05).

### Data Collection

We are grateful to Carlien Esterhuizen, Steve Dell and Declan Hofmeyr for providing den site locations for the MGR wild dog population. Wild dog research in the four study areas was generously supported by various organisations and individuals, which we gratefully acknowledge. Melanie Boshoff, Lynda Hedges, Herta van Helsdingen, Magriet van der Walt assisted with locating dens in VLNR. Wendy Collinson is gratefully acknowledged for initiating the idea and for useful discussions. Alistair Pole, Patrick Aust, Peter Lindsey and Stephanie Romanach are thanked for providing data on den site locations in SVC, as well as field scouts Rueben Bote and Misheck Matari. The Savé Valley Conservancy members kindly provided access to their properties. Support was received from the Zimbabwe Parks and Wildlife Management Authority and the Research Council of Zimbabwe granted permission to conduct the research. We are grateful to the Tanzania Wildlife Research Institute, De Beers Consolidated Mines and Jaguar Land Rover South Africa for support.

## Results

Wild dogs in all four populations consistently selected den sites in significantly more rugged terrain than what was available on average (ANOVA; MGR: H = 1038.142, df = 5, P = <0.001; VLNR: H = 243.549, df = 5, P = <0.001; LGCA: H = 938.108, df = 5, P = <0.001; SVC: H = 657.126, df = 5, P = <0.001). Within each study area, the multiple comparison indicated that TRI values around actual den sites were significantly greater (Dunn’s method, P = <0.05) than TRI values within equivalent distances from random contrast den sites.

**Figure 1:**
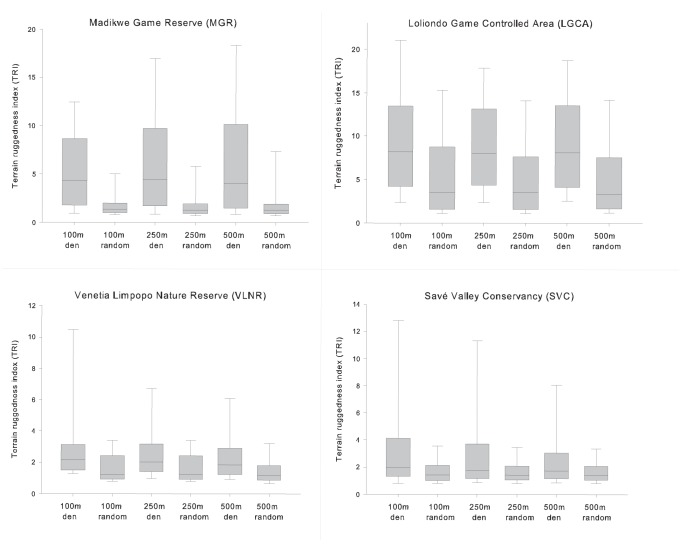
A boxplot showing the terrain ruggedness index values within 100 m, 250 m and 500 m of wild dog dens (“den”) and random contrast locations (“random”) within each of the four study populations. Median values are indicated in the boxes while the error bars indicate the 10^th^ and 90^th^ percentiles. Outliers not shown due to scaling of the graphs.

The analysis revealed that the LGCA is, on average, the most rugged and undulating study area (Fig. 1). The TRI values were also relatively high in MGR. However, rugged terrain in MGR occurs in the form of ridges and hills which stand out from relatively flat surroundings [30], while much of the LGCA is rugged and lacks large patches of even terrain. This is evident when contrasting the TRI values between the actual dens sites and random dens (Fig. 1). In MGR, random sites had extremely low TRI values relative to the actual dens sites. In LGCA the random sites are characterised by considerable ruggedness, yet the actual den sites are still located in significantly more rugged terrain.

Compared to LGCA and MGR, both VLNR and SVC had relatively low overall TRI values. The isolated nature and small size of rugged habitat patches in these study areas was also evident as the higher values, as indicated by the 90^th^ percentile error bar, decrease considerably with increasing distance from the den sites. In MGR, den sites were frequently situated in foothills of large ridge structures. Consequently, as the radii around den sites were increased from 100 m to 500 m, the skew in the data shifted to greater TRI values as a result of more of the rugged ridges being incorporated in the analysis.

## Discussion

Since young wild dogs are particularly vulnerable to predation, den site selection is a primary defence mechanism and strategy to maximise reproductive success. Our results clearly illustrate that rugged terrain is selected by wild dog packs for the purpose of reproduction and the rearing of pups. We postulate that this is due to risk avoidance, as hilly and mountainous terrain are not preferred habitat for lion or spotted hyaena [18], [20], and the probability of encountering these competitors are thus reduced in rugged terrain [20].

Wild dog packs accompanied by young pups are more likely to alarm call following exposure to lion roars than packs without young [16]. Despite their apparent increased sensitivity to lions’ presence, these packs were found to move shorter distances in the hour after hearing lions roaring as the young seemingly restricted their ability to rapidly vacate the immediate vicinity [16]. An area with low lion and spotted hyaena density, but with sufficient cover, would therefore be particularly favourable whilst raising pups [20]. In contrast to a direct encounter in open habitat, it is reasonable to assume that wild dog pups would be far less exposed in rugged terrain and have a greater chance of evading predators. Once the pups become older, they may be left alone while the pack goes out hunting, making such habitat even more important.

After leaving their pride to give birth, lionesses seek dense cover to rear their cubs and may utilise hills [31]. However, they use the habitat for this purpose alone and may be disinclined to engage in any risk-prone interactions with other predators during this vulnerable time. Their use of similar rugged habitat for reproduction also indicates the potential safety that these habitats may afford vulnerable altricial offspring. Furthermore, since lionesses with young are the only portion of the lion population that periodically seeks out and utilises rugged habitat, it further substantiates the argument that these habitats are generally safer, with reduced levels of inter- and intra-specific competition.

### Implications for Conservation Planning

Wild dogs have evolved behavioural mechanisms that allow them to occur sympatrically with dominant carnivores. These mechanisms evolved in large heterogeneous landscapes where the density and distribution of competitors varied spatially and temporally. Wild dogs are particularly prone to anthropogenic threats, and protected areas are therefore of importance for their survival [32]. However, interspecific competition within protected areas may significantly inhibit conservation efforts for this endangered carnivore [33]. An understanding of competition refuges [34], especially for reproduction, is thus of great relevance as these may be limited in relatively small protected areas. A simple analysis of terrain ruggedness would rapidly determine the extent of potential denning habitat within a given protected area, thereby assisting assessments of its suitability to conserve wild dogs. While it has been suggested that wild dog reintroductions should not be conducted in reserves with high lion densities [35]–[36], landscape heterogeneity may largely determine the degree to which the two species can coexist. A case in point is Pilanesberg National Park (PNP) in South Africa. Despite the small 572 km^2^ reserve’s lion population exceeding 140 lions at times, wild dogs have survived on the reserve since their reintroduction in 1999 [20]. PNP is located in an inactive volcanic crater and the reserve’s topography is characteristically rugged. Dens were located in rugged areas that were known to be low lion density areas and, more specifically, lions were “not known to use the steep slopes… where dens were located” [20]. An analysis of mean terrain ruggedness values within ten protected areas with resident wild dog populations revealed that PNP had the greatest TRI values (Jackson et al., unpublished data). Although LGCA had the greatest TRI values in this study, the mean terrain ruggedness in PNP is twice that of LGCA. Similarly, reintroduced wild dogs in the characteristically rugged Hluhluwe-iMfolozi Park, South Africa, have also fared well despite the presence of sizeable lion and spotted hyaena populations [37]. Evidence therefore suggests that undulating terrain plays an important role in wild dogs’ spatial avoidance of lions thereby facilitating sympatric coexistence of wild dogs and lions. Consequently, an assessment of topographic heterogeneity may be important in wild dog conservation planning, particularly during habitat assessments prior to potential reintroductions.

Here we have indicated, for the first time, the selection of rugged terrain for denning purposes in four populations of wild dogs, using a novel technique that has significant conservation applications. The simple technique used to devise the terrain ruggedness index may be useful to investigate habitat suitability, and even predict highly suitable denning areas, across large landscapes. The technique may also be applicable in the studies of other carnivores which have a strong association with rugged terrain, such as leopards (*Panthera pardus*; [38]). Future work may aim to establish the relationship between lion and wild dog densities at finer spatial resolutions and establish how the availability of rugged terrain may influence interspecific coexistence and relative densities of the two species.
